# Gendist: An R Package for Generated Probability Distribution Models

**DOI:** 10.1371/journal.pone.0156537

**Published:** 2016-06-07

**Authors:** Shaiful Anuar Abu Bakar, Saralees Nadarajah, Zahrul Azmir ABSL Kamarul Adzhar, Ibrahim Mohamed

**Affiliations:** 1 Institute of Mathematical Sciences, University of Malaya, Kuala Lumpur, Malaysia; 2 School of Mathematics, University of Manchester, Manchester, United Kingdom; Fondazione Edmund Mach, Research and Innovation Centre, ITALY

## Abstract

In this paper, we introduce the R package gendist that computes the probability density function, the cumulative distribution function, the quantile function and generates random values for several generated probability distribution models including the mixture model, the composite model, the folded model, the skewed symmetric model and the arc tan model. These models are extensively used in the literature and the R functions provided here are flexible enough to accommodate various univariate distributions found in other R packages. We also show its applications in graphing, estimation, simulation and risk measurements.

## Introduction

Various probability distribution models have been proposed in the past and the number increases with time. Recently, in the area of actuarial loss modeling, several new models were found to provide good fits to the loss data. For instance, mixture of Erlang distribution has been proposed to model catastrophic loss data in the United States [1]. Mixture of exponential with peaks-over-threshold has been considered to fit Danish fire losses and medical claims data found in Society of Actuaries (SOA) Group Medical Large Claims Database [2]. Mixture of lognormal and inverse Gaussian distribution were used to model fire insurance portfolio in Serbia [3]. In the actuarial context, loss data are monetary losses claimed by insureds purchasing general insurance policies such as fire and catastrophic insurances. Many reasons in which the data is found to be crucial and recorded by insurance companies or insurance service agencies, among others, to model their future financial obligations. In addition to the above, various applications of mixture model can be found in the literature for other area of studies. To name a few: a logistic mixture distribution model has been applied on polychotomous item responses [4]; mixture of logistic has been proposed to fit long tail distributions in analyzing network performance [5]; Rayleigh mixture model has been studied for plaque characterization in intravascular ultrasound [6]; a finite mixture of two Weibull distributions has been suggested to model the diameter distributions of rotated-sigmoid, uneven-aged stands [7]; two-component mixture Weibull statistics has been used to estimate wind speed distributions [8]; gamma mixture models have been proposed for target recognition [9]; Gaussian mixture model has been applied for human skin color and its applications in image and video databases [10].

Another important model receiving increasing applications in actuarial loss modeling is the composite model. Composite model is constructed by piecing two weighted distributions together. Such a model has been introduced initially with constant mixing weight [11]. It was then improved by allowing flexible mixing weight [12]. Several other related composite models have been proposed by various authors, see [13–15] and [16]. All these authors employed the well known Danish fire insurance data to measure their model performance. Composite models have also been applied to some simulated data belong to a particular class of distribution. Among others, a comparison study have been made on Weibull-Pareto and Lognormal-Pareto composite models [17]. Besides, some properties, inferences and numerical illustration using simulated data have been provided for composite exponential-Pareto models [18].

Another model that provides useful application in loss modeling is the folded model. It has been used to model the Norwegian fire claims data [19]. An extension to this study proposed three new folded models, namely, the folded generalized *t* distribution, folded Gumbel distribution and folded exponential power distribution [20]. An interesting feature of this model is the folding mechanism of a real value defined distribution into positive value distribution. Several existing folded models found in the literature are the folded normal distribution [21], folded *t* distribution [22], folded Cauchy distribution [23] and folded logistic distribution [24].

In addition to the above, skewed symmetric models also featured attractive properties with respect to loss data. The skewed normal and skewed *t* distributions have been studied in fitting insurance claims data [25]. Later, the same author applied the two models to asset returns of insurance companies [26]. Skewed *t* distribution is found to provide promising results for both data. A variety of other skew models have been considered, among them, skewed Cauchy [27], skewed Laplace [28], skewed logistic [29], skew reflected gamma, skew double Weibull and skew beta-prime [30] and several skew inverse reflected distributions [31].

More recently, the arc tan model has been introduced to model Norwegian fire claims data [32]. This new methodology has been proposed for an underlying Pareto distribution and found to provide good fit compared to several classical distributions. Its statistical properties also have been derived.

Beside heavily used in the actuarial field, most of the aforementioned models received vast applications in many other areas. Motivated by this, we compile several important models into an R package **gendist** for academics and public use. R is a free statistical computing and graphics software downloadable from http://www.r-project.org, see [33].

Distribution models provided in the R package **gendist** include the mixture, the composite, the folded, the skewed symmetric and the arc tan models. Computation functions of these models are given for probability density function (pdf), cumulative distribution function (cdf), quantile function (qf) and random generated values (rg).

The conventional R prefixes d, p, q and r define the pdf, cdf, qf and rg of an arbitrary distribution function. For instance dexp, pexp, qexp and rexp of the **stats** package [33] in R gives the pdf, cdf, qf and rg for an exponential distribution. The **gendist** package follows similar rule to define all the functions related to the generated probability distribution models.

In preparing the **gendist** package, we have thoroughly studied the Distribution CRAN Task View of R [34] which lists a number of available packages related to probability distributions. None of the packages on this page has so far provide tools to work with the folded and arc tan models discussed in this paper. Several packages are found for mixture models: **mixtool** [35] provides d and r functions for finite mixture models; **nor1mix** [36] provides d, p and r functions for a specific underlying distribution, Gaussian; **GSM** [37] provides d, p and r functions with underlying Gamma shape distribution; **AdMit** [38] provides d and r functions for mixture of student distribution.

Only the **CompLognormal** package [39] is available for composite models. However, the model is restricted to head of a lognormal distribution. In addition, the function is bound below at zero. For skewed symmetric model, several packages are available: **skewt** [40], **sn** [41], **gamlss.dist** [42] and **sgt** [43] provides functions d, p, q and r for variety of either skew normal or skew student distributions; **Newdistns** [44] provides functions related to skew symmetric G distribution. **SkewHyperbolic** [45] provides functions related to skew hyperbolic student distribution. All the distributions mentioned above plus others (including newly proposed distributions) can be easily managed by the functions developed in the proposed **gendist** package.

The paper is structured as follows. First, we discuss in detail all the above models along with examples on using related functions developed in the **gendist** package. Then, we provide descriptions on the program structure of the package and some discussion on appropriate usage with respect to the support of the models. We finally conclude the works carried out. Further details of the **gendist** package can be found at http://CRAN.R-project.org/package=gendist.

## Generated Probability Distribution Models

Models presented here are generated with underlying parent distributions. These models are specified by a predefined rule or structure. In what follows, we describe in detail each generated model giving particular emphasis on related functions encompassed in the **gendist** package.

### The mixture models

The mixture model was first considered with an underlying normal distribution to address the decomposition issue with respect to non-normal forehead to body length attributes in a population of female shore crabs, see [46]. In general, the pdf of the mixture model is given by

$$\begin{array}{c} f\left(x\right)=\sum_{i=1}^{n}w_{i}g_{i}\left(x\right) \end{array}$$ (1)

where 0 ≤ *w*_*i*_ ≤ 1 for *i* = 1, 2, …, *n* are the mixing weights such that the sum of all *w*_*i*_ equals to one. Note that, there are *n* components involved in Eq (1).

The proposed mixture model in this paper is restricted to a two component distribution. Hence, the pdf can be represented as follows:

$$\begin{array}{c} f\left(x\right)=wg_{1}\left(x\right)+\left(1-w\right)g_{2}\left(x\right) \end{array}$$ (2)

We choose mixing weight of the form $w=\frac{1}{1+\phi }$ so that *ϕ* > 0. *g*_1_(*x*) and *g*_2_(*x*) represent the densities of arbitrary parent distributions. Note, it is not necessary that the two arbitrary distributions in Eq (2) are identical. However, both must be defined on the same range dimension.

Finding cdf of the mixture model is straightforward. By direct integration, the cdf for the two component mixture model in Eq (2) can be written as

$$\begin{array}{c} F\left(x\right)=\frac{1}{1+\phi }G_{1}\left(x\right)+\frac{\phi }{1+\phi }G_{2}\left(x\right) \end{array}$$ (3)

with *G*_*i*_(*x*) for *i* = 1, 2 are arbitrary cdfs of the parent distribution.

Explicit general expression for qf of the mixture model is not available. However, it can be obtained numerically by solving the root, *Q*(*u*), of the following equation

$$\begin{array}{c} wG_{1}\left(Q\left(u\right)\right)+\left(1-w\right)G_{2}\left(Q\left(u\right)\right)-u=0 \end{array}$$ (4)

Finally, random generated numbers for the two component mixture model can be obtained as

$$\begin{array}{c} x_{i}=Q\left(u_{i}\right) \end{array}$$ (5)

where *u*_*i*_ for *i* = 1, 2, ⋯, *n* are random numbers from a uniform [0, 1] distribution.

In what follows, we show an application of the cdf function of the mixture model, pmixt to measure the closeness of two different set of measurements using P-P plot. Data used for this purpose is the US indemnity loss data scaled by 1000 found in the **copula** package [47]. We also use the **actuar** package [48] to illustrate several heavy tailed distributions as theoretical measurements. Assuming pre-installation of both packages the data (S1 Data) can be obtained and sorted as follows:

R> library(actuar)

R> library(copula)

R> data(loss)

R> x  <- loss$loss/1000

R> n  <- length(x)

R> xx  <- seq(1, n)/(n+1)

R> x  <- sort(x)

For each distribution involved, values of 0.2, 0.7 and 20 are chosen for phi (the mixing weight component), shape and scale parameters, respectively. These values are only for illustration purposes. The P-P plots are produced by the following command:

R> par(mfrow = c(2, 2))

R> arg1 <- c(shape = 0.7, scale = 20)

R> arg2 <- c(shape = 0.7, scale = 20)

R> plot(xx, pmixt(x, phi = 0.2, spec1 = “weibull”, arg1,

+   spec2 = “llogis”, arg2), xlim = c(0,1), ylim = c(0, 1), col = 2,

+   ylab = “Expected”, xlab = “Observed”,

+   main = “Mixture of Weibull-Loglogistic”)

R> abline(0, 1)

R> plot(xx, pmixt(x, phi = 0.2, spec1 = “weibull”, arg1,

+   spec2 = “pareto”, arg2), xlim = c(0,1), ylim = c(0,1), col = 2,

+   ylab = “Expected”, xlab = “Observed”,

+   main = “Mixture of Weibull-Pareto”)

R> abline(0,1)

R> plot(xx, pmixt(x, phi = 0.2, spec1 = “weibull”, arg1,

+   spec2 = “invpareto”, arg2), xlim = c(0,1), ylim = c(0,1), col = 2,

+   ylab = “Expected”, xlab = “Observed”,

+   main = “Mixture of Weibull-Inverse Pareto”)

R> abline(0,1)

R> plot(xx, pmixt(x, phi = 0.2, spec1 = “weibull”, arg1,

+   spec2 = “paralogis”, arg2), xlim = c(0,1), ylim = c(0,1), col = 2,

+   ylab = “Expected”, xlab = “Observed”,

+   main = “Mixture of Weibull-Paralogistic”)

R> abline(0,1)

#### Analysis of Application on Mixture Model

**Fig 1:**
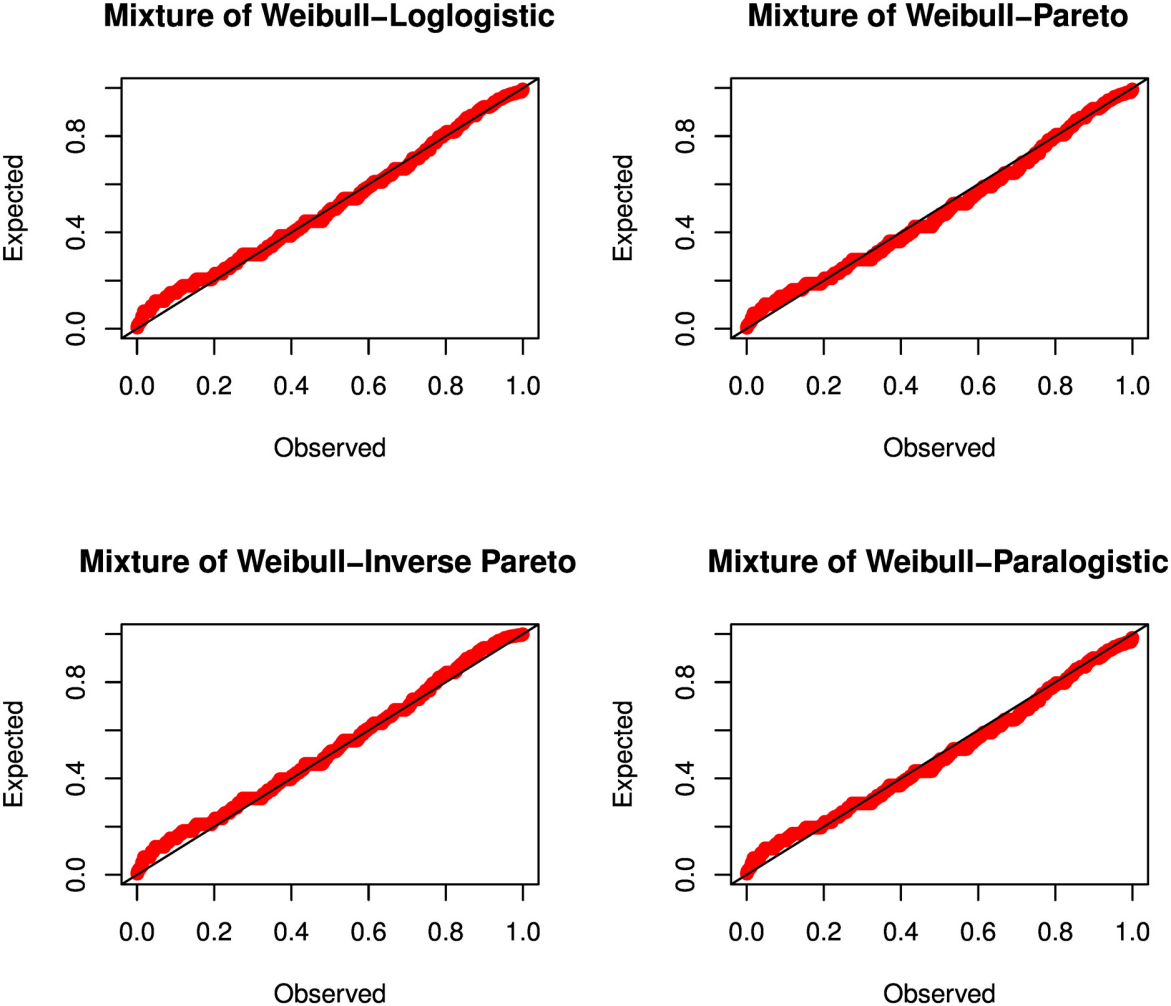
Probability-probability plots (P-P plots) for the US indemnity loss data for several mixture models. Expected and observed denote the theoretical and empirical data, respectively. Four models are shown including the mixture of Weibull-Logistic, mixture of Weibull-Pareto, mixture of Weibull-Inverse Pareto and mixture of Weibull-Paralogistic models. Values of 0.2, 0.7 and 20 are chosen for the mixing weight component, shape and scale parameters. All the models show a good fit to the empirical data.

P-P plots are used to compare the degree of agreement between two sets of measurements based on their cdf. Fig 1 shows the P-P plot of the empirical data against the theoretical values of the mixture of Weibull-Loglogistic, Weibull-Pareto, Weibull-Inverse Pareto and Weibull-Paralogistic models. A small deviation of the plots to the 45° line indicate the closeness of the empirical and theoretical probability and vice versa. It is apparent from the P-P plots that the fits are good for all the theoretical models proposed.

### The composite models

In general, the pdf of the composite model is given by

$$\begin{array}{c} f\left(x\right)=\left\{\begin{array}{cc} wf_{1}^{*}\left(x\right), & \text{if}\;0<x\leq \theta \\ \left(1-w\right)f_{2}^{*}\left(x\right), & \text{if}\;\theta <x<\infty \end{array}\right. \end{array}$$ (6)

whereby *w* is the mixing weight, *θ* denote the threshold and $f_{i}^{*}\left(x\right)$ for *i* = 1, 2 are the truncated pdfs of the parent distribution defined by

$$\begin{array}{c} f_{1}^{*}\left(x\right)=\frac{f_{1}\left(x\right)}{F_{1}\left(x\right)} \end{array}$$ (7)

and

$$\begin{array}{c} f_{2}^{*}\left(x\right)=\frac{f_{2}\left(x\right)}{1-F_{2}\left(x\right)} \end{array}$$ (8)

respectively. Eq (6) can be made continuous and smooth by applying the continuity and differentiability conditions and thus provide a closed form for *w*, see [12]. Furthermore, it has been suggested that *w* take the form of $w=\frac{1}{1+\phi }$ for *ϕ* > 0 for convenience [13]. We find this useful to ease program writing in R for the composite model. A more comprehensive approach to implement Eq (6) which we adopt in this paper specifies the component of mixing weight, *ϕ*, and the threshold, *θ* in term of other parameters of the model, see [15]. Further to this, we do not restrict the composite model to bound below at zero and thus allow parent distribution defined on real line as well as that defined for positive values to be considered for $f_{1}^{*}\left(x\right)$. All other functions including the cdf, qf, and rg for the composite model are developed in a similar manner.

Hence, the cdf, qf and *n* random generated numbers provided in **gendist** package can be written as

$$\begin{array}{c} F\left(x\right)=\left\{\begin{array}{cc} \frac{1}{1+\phi }\frac{F_{1}\left(x\right)}{F_{1}\left(\theta \right)} & \text{if}\;\;x\leq \theta \\ \frac{1}{1+\phi }\left(1+\phi \frac{F_{2}\left(x\right)-F_{2}\left(\theta \right)}{1-F_{2}\left(\theta \right)}\right) & \text{if}\;\;x>\theta \end{array}\right. \end{array}$$ (9)

$$\begin{array}{c} \begin{array}{c} Q\left(u\right)=\left\{\begin{array}{cc} Q_{1}^{*}\left(u\left(1+\phi \right)F_{1}\left(\theta \right)\right) & \text{if}\;\;u\leq \frac{1}{1+\phi }\\ Q_{2}\left(F_{2}\left(\theta \right)+\left(1-F_{2}\left(\theta \right)\right)\left(\frac{u(1+\phi )-1}{\phi }\right)\right) & \text{if}\;\;u>\frac{1}{1+\phi } \end{array}\right. \end{array} \end{array}$$ (10)

and

$$\begin{array}{c} x_{i}=Q\left(u_{i}\right) \end{array}$$ (11)

where *u*_*i*_ with *i* = 1, 2, …, *n* are *n* uniform [0, 1] random numbers.

As a result, the number of parameters of the composite models equal the total number of parameters of the two underlying parent distributions selected. For instance, if truncated exponential distribution with scale parameter is chosen for $f_{1}^{*}\left(x\right)$ and Weibull distribution with scale and shape parameters are chosen for $f_{2}^{*}\left(x\right)$, the Exponential-Weibull composite model will consist of three parameters.

We now show the implementation of the pdf function dcomposite of the composite model. All other functions related to the composite model follow similarly. Let *f*_1_(*x*) and *f*_2_(*x*) denote the pdfs of Weibull and Gamma distributions, respectively. From Eq (6) (with $w=\frac{1}{1+\phi }$), the composite Weibull-Gamma model can be written as

$$\begin{array}{c} f\left(x\right)=\left\{\begin{array}{cc} \frac{1}{1+\phi }\frac{\beta e^{-{\left(\frac{x}{\lambda }\right)}^{\beta }}{\left(\frac{x}{\lambda }\right)}^{\beta }}{x-xe^{-{\left(\frac{\theta }{\lambda }\right)}^{\beta }}}, & \text{if}\;0<x\leq \theta \\ \frac{\phi }{1+\phi }\frac{\sigma^{-\alpha }x^{\alpha -1}e^{-\frac{x}{\sigma }}}{\Gamma \left(\alpha ,\frac{\theta }{\sigma }\right)}, & \text{if}\;\theta <x<\infty \end{array}\right. \end{array}$$ (12)

where Γ(*a*, *z*) is the incomplete gamma function defined by $\int_{z}^{\infty }t^{\alpha -1}e^{-t}dt$. Suppose, *α* = *β* = 1 and *σ* = *λ* = 2, then, the pdf values of the composite model for some values of *x* are computed as follows:

R> dcomposite(1:3, spec1 = “weibull”, arg1 = list(shape = 1, scale = 2), spec2 = “gamma”, arg2 = list(shape = 1, scale = 2))

Output:

0.3032653 0.1839397 0.1115651

Density plots of this model can be done using curve function as below.

R> curve(dcomposite(x, spec1 = “weibull”, arg1 = list(shape = 2.5, scale = 1),

+    spec2 = “gamma”, arg2 = list(shape = 1, scale = 1), initial = c(0.1, 1)),

+    xlim = c(0,10), xlab = “x”, ylab = “f(x)”)

R> curve(dcomposite(x, spec1 = “weibull”, arg1 = list(shape = 1.5, scale = 2),

+    spec2 = “gamma”, arg2 = list(shape = 2, scale = 1), initial = c(0.1, 2)),

+    add = TRUE, col = 2)

R> curve(dcomposite(x, spec1 = “weibull”, arg1 = list(shape = 2.3, scale = 2),

+    spec2 = “gamma”, arg2 = list(shape = 2, scale = 0.5),

+    initial = c(0.1,10)), add = TRUE, col = 3)

R> curve(dcomposite(x, spec1 = “weibull”, arg1 = list(shape = 0.1, scale = 2),

+    spec2 = “gamma”, arg2 = list(shape = 0.5, scale = 1),

+    initial = c(0.1,10)), add = TRUE, col = 4)

#### Analysis of Application on Composite Model

**Fig 2:**
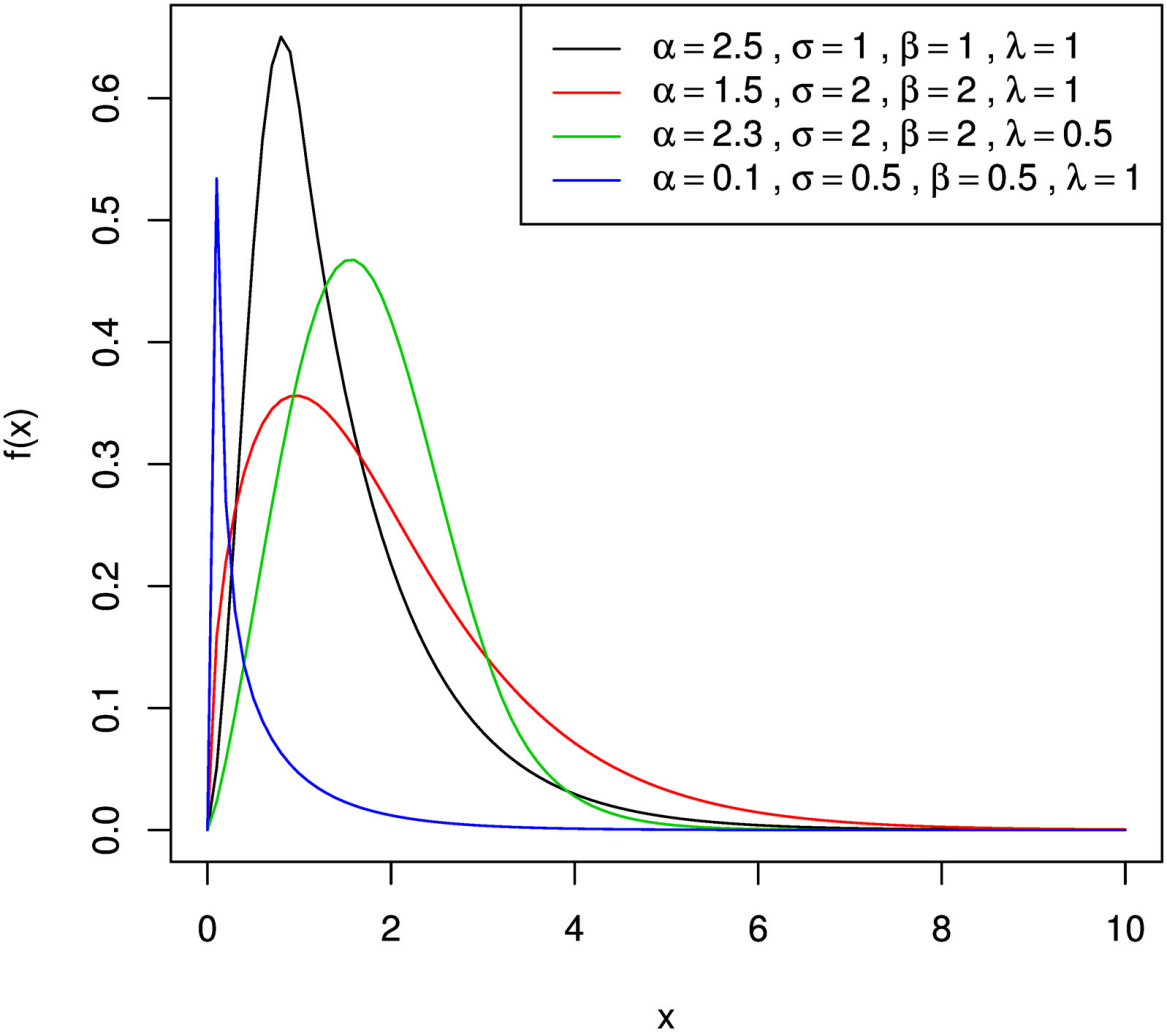
Probability density function curves for composite Weibull-Gamma model with varying parameters. Four composite Weibull-Gamma models with varying parameters are shown. The shapes depend on the value of parameters chosen.

Fig 2 shows the pdf plots of the composite Weibull-Gamma models with varying parameter values. The density shapes depend on the values of the scale and shape parameters. For the composite Weibull-Gamma model, all the four densities exhibit unimodal distribution, that is, they have a single mode. It is also observed that choosing lower shape parameters for both parent distributions will result in a thin and steep distribution while having a larger value result in a thick and gradual slope. Note that the observation made here applies for composite Weibull-Gamma. Other composite models may have different result depending on the features of the pair of the parent distributions chosen.

### The folded models

Existence of folded models can be traced back to 1960s when methods were described to estimate mean and variance of normal distribution based on its folded form and an application was shown to real camber data [21]. The folded model is obtained from a transformation of random variable by taking its absolute value. Suppose that *Y* is a real valued random variable with cdf *G*(⋅) and *X* = |*Y*| a positive random variable, then the cdf for *X* is

$$\begin{array}{cc} F(x)=G(x)-G(-x) & \;x>0 \end{array}$$ (13)

We can then write its pdf as

$$\begin{array}{cc} f(x)=g(x)+g(-x) & \;x>0 \end{array}$$ (14)

In this case the parent distribution has a support of real values and the resulted folded distribution has *x* > 0.

It is obvious from Eq (13) that the quantile function may not have an explicit form. However, this may not be the case if the underlying distribution is symmetric around 0. The quantile function for a folded model of this case can be obtained as follows:

$$\begin{array}{c} Q\left(u\right)=F^{-1}\left(u\right)=G^{-1}\left(\frac{1+u}{2}\right) \end{array}$$ (15)

where *G*^−1^(⋅) is the inverse function of the underlying symmetric distribution. For an underlying non-symmetric distribution (also applies to symmetric case), we can solve this numerically using computer programs by finding root of the cdf function, that is, *Q*(*u*) is the solution of the following equation

$$\begin{array}{c} G(Q(u))-G(-Q(u))-u=0 \end{array}$$ (16)

Next, rg is obtained as follows

$$\begin{array}{c} x_{i}=Q\left(u_{i}\right) \end{array}$$ (17)

where *u*_*i*_ with *i* = 1, 2, …, *n* are *n* uniform [0, 1] random numbers. Note that *g*(⋅) and *G*(⋅) correspond to the pdf and cdf of the parent distribution of the folded model. We implement Eqs (13), (14), (16) and (17) in the **gendist** package for cdf, pdf, qf and rg of the folded model.

In what follows, we show applications of two risk measures by implementation of qf for the folded model, qfolded. It involves computation of Value-at-Risk (VaR) and Conditional Tail Expectation (CTE). VaR is related to the quantile function of a random variable with a specified probability, say, *α*. Often it describes the risk that a loss random variable exceeds a certain amount although it is also used outside the financial area. Let *G*^−1^(*α*) denote the inverse function of *G*(*x*) for a random variable *Y*, then VaR at a given level of confidence, 1 − *α*, is given by

$$\begin{array}{c} VaR\left(\alpha \right)=G^{-1}\left(1-\frac{\alpha }{2}\right) \end{array}$$ (18)

**Fig 3:**
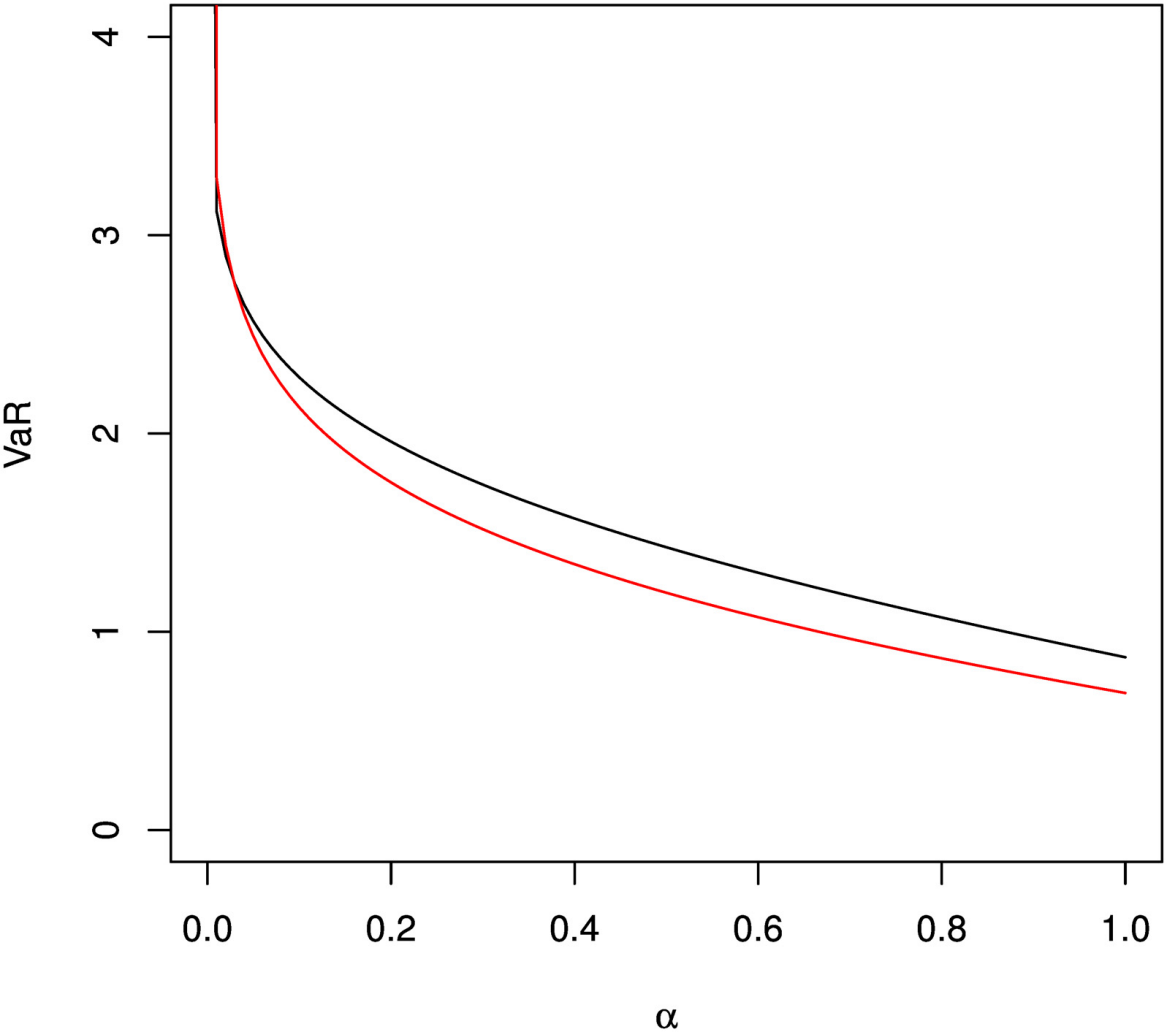
Value-at-Risk for folded normal (black) and folded t (red) distributions.

For a range of *α* values, this is illustrated for folded normal and folded *t* distributions in Fig 3. Some arbitrary values are chosen for the parameters of both models. The following commands describe the plotting of VaR with respect to *α*:

R> curve(qfolded(1-x/2, spec = “norm”, arg = c(mean = 0.8, sd = 0.9),

+    interval = c(0,100)), xlim = c(0,1), ylim = c(0,4),

+    xlab = expression(alpha), ylab = “VaR”)

R> curve(qfolded(1-x/2, spec = “t”, arg = c(df = 15), interval = c(0,100)),

+    add = TRUE, col = 2)

The CTE measures the expected value of risk beyond VaR at a given probability level, *α*. Mathematically,

$$\begin{array}{c} CTE\left(\alpha \right)=\frac{1}{\alpha }\int_{\alpha }^{1}G_{Y}^{-1}\left(1-\frac{\alpha }{2}\right)d\alpha \end{array}$$ (19)

**Fig 4:**
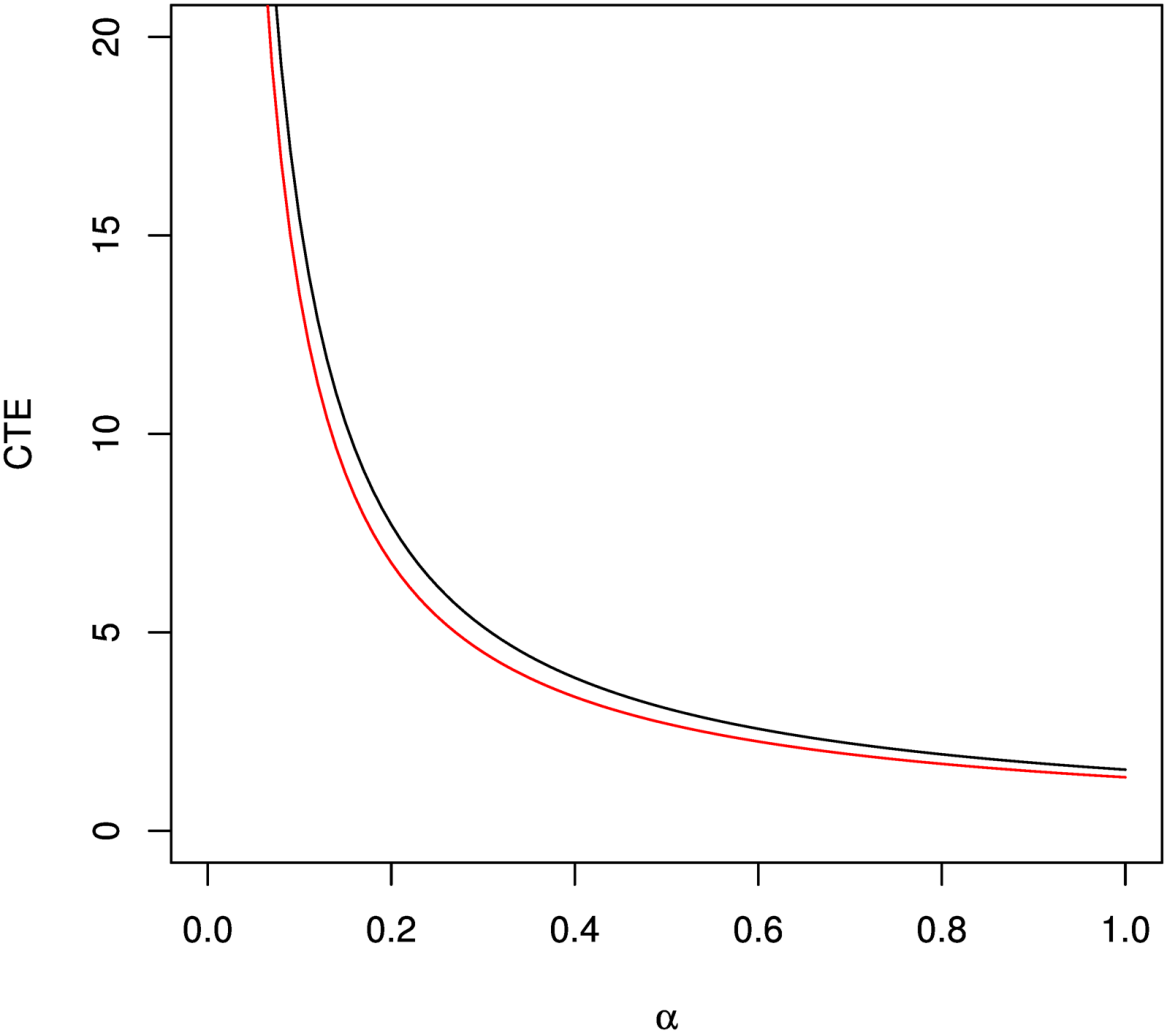
Conditional Tail Expectation for folded normal (black) and folded t (red) distributions.

For a range of *α* values, this is illustrated for folded normal and folded *t* distributions in Fig 4. The following commands describe the plotting of CTE with respect to *α*:

R> curve((1/(x))*integrate(function(x)qfolded(1-x/2, spec = “norm”,

+    arg = c(mean = 0.8, sd = 0.9), interval = c(0,100)), x, 1)\$value,

+    xlim = c(0,1), ylim = c(0,20), xlab = expression(alpha), ylab = “CTE”)

R> curve((1/(x))*integrate(function(x)qfolded(1-x/2, spec = “t”,

+    arg = c(df = 15), interval = c(0,100)), x, 1)\$value, add = TRUE, col = 2)

#### Analysis of Application on Folded Model

Fig 3 shows that for both distributions VaR appears to be a decreasing function of the probability level *α*. This is a natural behavior of risk, that is, higher VaR is found for higher level of confidence. In practice, *α* are commonly chosen as 1% or 5%. In this example, folded *t* distribution consistently show a lower VaR value than the normal distribution. However, the actual results will depend on the parameter values for each model. Similar to VaR, CTE for both distributions are decreasing function of the confidence level, *α*. This is depicted in Fig 4.

### The skewed symmetric models

Azzalini proposed a new class of distributions with an underlying normal distribution [49]. In its original form, the pdf is given by

$$\begin{array}{c} f(x)=2g(x)G(\lambda x),\;-\infty <x<\infty \end{array}$$ (20)

where *g*(⋅) and *G*(⋅) are the pdf and cdf of the normal distribution and *λ* is a shape parameter. The distribution reduces to normal distribution when *λ* = 0.

Further study leads to a general form of the skew symmetric distribution [50]. The new skewed symmetric distribution has the following pdf

$$\begin{array}{cc} f(x)=2h(x)G(x), & -\infty <x<\infty \end{array}$$ (21)

where *h*(*x*) is a pdf symmetric at 0 and *G*(*x*) is a Lebesgue measurable function that satisfies 0 ≤ *G*(*x*) ≤ 1 and *G*(*x*) + *G*(−*x*) = 1 for *z* ∈ ℜ. In this new form, the parent distributions *h*(*x*) and *G*(*x*) may be of different type satisfying the conditions above. In the **gendist** package, we adopt Eq (21) for computer implementation of the skewed symmetric model. Several functions for *h*(*x*) and *G*(*x*) include the normal, logistic, students *t* and Cauchy distributions.

General form for the cdf, qf and rg functions of the skewed symmetric models are not available. Thus, implementation of these functions in **gendist** are done via numerical methods. In particular, integrate and uniroot functions are used. The cdf of skewed symmetric model is found by

$$\begin{array}{cc} F\left(x\right)=\int_{-\infty }^{x}2h\left(y\right)G\left(y\right)dy, & -\infty <x<\infty \end{array}$$ (22)

Solving *Q*(*u*) of the following equation leads to its qf

$$\begin{array}{c} \int_{-\infty }^{Q(u)}2h\left(y\right)G\left(y\right)dy-u=0 \end{array}$$ (23)

and rg is obtained as follows

$$\begin{array}{c} x_{i}=Q\left(u_{i}\right) \end{array}$$ (24)

where *u*_*i*_ with *i* = 1, 2, …, *n* are *n* uniform [0, 1] random numbers.

Some skewed symmetric models may have an explicit form. For instance, consider a skewed Cauchy-Cauchy distribution. Its pdf, cdf and qf are given by

$$\begin{array}{c} f\left(x\right)=\frac{\lambda \left(2\tan^{-1}\left(\frac{x}{\lambda }\right)+\pi \right)}{\pi^{2}\left(\lambda^{2}+x^{2}\right)} \end{array}$$ (25)

$$F(x)=\frac{{\left(2{\text{ tan}}^{-1}\left(\frac{x}{\lambda }\right)+\pi \right)}^{2}}{4\pi^{2}}$$ (26)

and

$$Q(u)=\lambda \text{ tan}\left(\frac{1}{2}\left(2\pi \sqrt{u}-\pi \right)\right)$$ (27)

respectively.

In the following illustration, we check goodness-of-fit of two theoretical distributions, the skew Logistic-Logistic and the skew Normal-Normal distributions, to the length of rivers scaled by 1000. The data consist of 141 observations related to major North American rivers. The data (S2 Data) can be obtained as follows:

R> data(rivers)

R> x <- rivers/1000

Initial step involves parameter estimation using the maximum likelihood method.

R> nloglik <- function(p, spec1, arg1, spec2, arg2){

+ tt  <- 1.0e20

+ if(all(p>0)){

+  tt  <- -sum(log(dskew(x, spec1, arg1, spec2, arg2)))

+ }

+ return(tt)

+ }

R> par <- nlm(function(p){nloglik(p,spec1 = “logis”, arg1 = list(scale = p [1]),

+   spec2 = “logis”, arg2 = list(scale = p [2])), p = c(1,1)) $estimate

R> par2<- nlm(function(p){nloglik(p, spec1 = “norm”, arg1 = list(sd = p [1]),

+   spec2 = “norm”, arg2 = list(sd = p [2]))}, p = c(1,1)) $estimate

**Fig 5:**
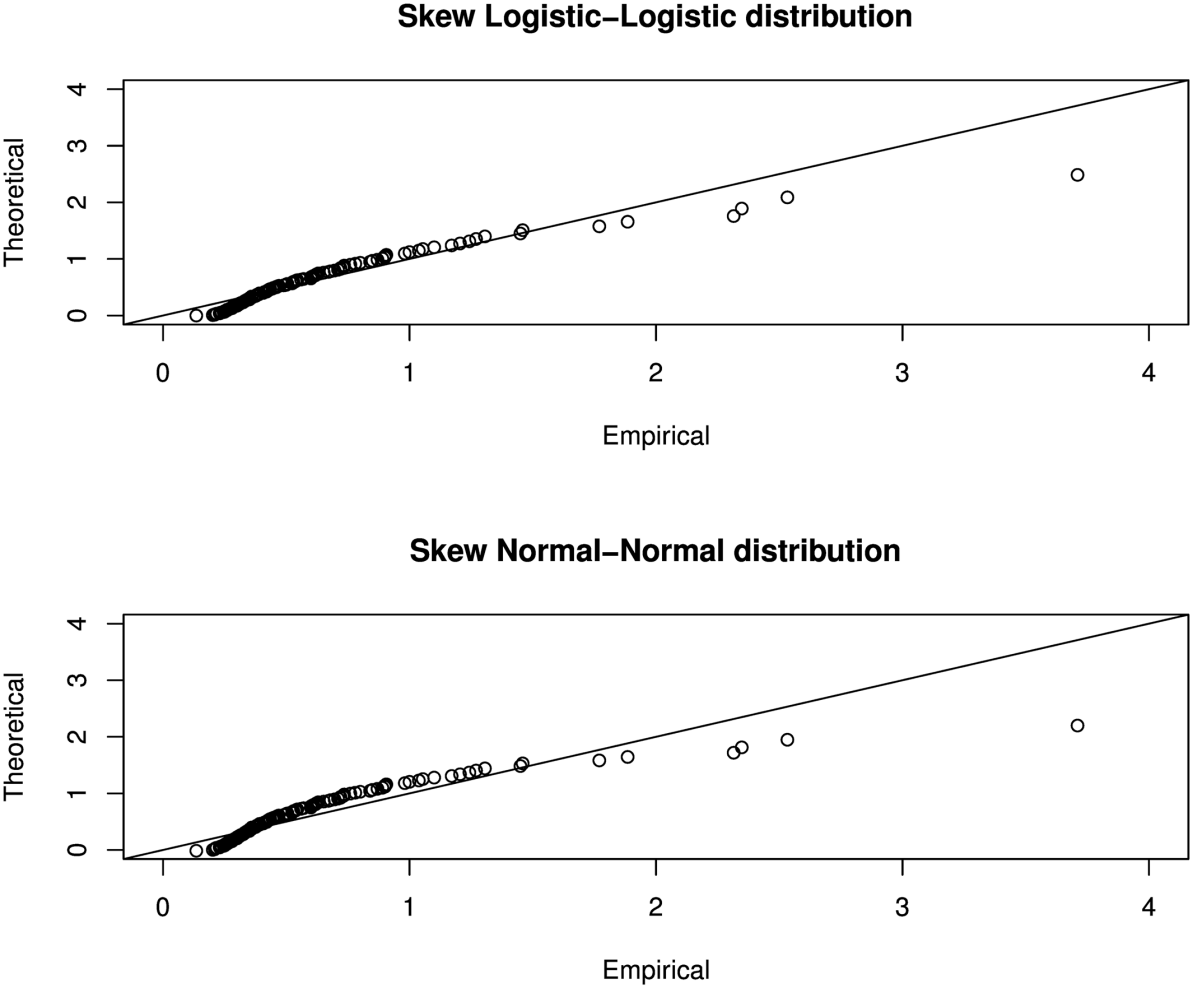
Q-Q plots of rivers data for the fitted skew logistic-logistic and skew normal-normal distributions. Empirical values are plotted against two theoretical distributions, that is, the skew Logistic-Logistic distribution and the skew Normal-Normal distribution. Both show a close plot to the 45° line except for several extreme values.

Finally, the Q-Q plots are produced as presented in Fig 5 with the following commands:

R> qqplot(x, qskew(u, spec1 = “logis”, arg1 = list(scale = par [1]),

+    spec2 = “logis”, arg2 = list(scale = par [2]), interval = c(-10,10)),

+    xlim = c(0,4), ylim = c(0,4), ylab = “Theoretical”, xlab = “Empirical”,

+    main = “Skew Logistic-Logistic distribution”)

R> abline(0,1)

R> qqplot(x,qskew(u, spec1 = “norm”, arg1 = list(sd = par2 [1]), spec2 = “norm”,

+    arg2 = list(sd = par2 [2]), interval = c(-10,10)), xlim = c(0,4),

+    ylim = c(0,4), ylab = “Theoretical”, xlab = “Empirical”,

+    main = “Skew Normal-Normal distribution”)

R> abline(0,1)

#### Analysis of Application on Skewed Symmetric Model

Parameter estimation is performed using the maximum likelihood method which is known to have the following properties: sufficient, invariance, consistent, efficient and asymptotically normal. Estimated parameters are then used to find the theoretical values of the quantile function. These values are matched to the empirical values and plotted to the Q-Q plot. Goodness-of-fit is then measured by relative distance of the plots to the 45° line. Closer theoretical versus empirical plots to the line indicate a good fit and vice versa. Majority of the plots in Fig 5 are close to the line except for some values at the extreme ends. These are the five longest river in the North America. Between the two models, the skew Logistic-Logistic distribution gives a lower negative log likelihood value, that is, a value of 57.5903. Thus it has a better goodness-of-fit to the river data as compared to the skew Normal-Normal distribution.

### The arc tan models

Arc tan model has been proposed to model a specific Norwegian insurance loss data [32]. Consider parent distribution with pdf and cdf of *g*(*x*) and *G*(*x*), respectively. Then, the pdf, cdf, qf and rg of the arc tan model with support [*a*, *b*] are given by

$$\begin{array}{c} f\left(x\right)=\frac{1}{\arctan(\alpha )}\frac{\alpha g(x)}{1+{\left(\alpha (1-G(x))\right)}^{2}} \end{array}$$ (28)

$$\begin{array}{c} F\left(x\right)=1-\frac{\arctan(\alpha (1-G(x)))}{\arctan(\alpha )} \end{array}$$ (29)

$$\begin{array}{c} Q\left(u\right)=G^{-1}\left(1-\frac{1}{\alpha }\tan\left(\left(1-u\right)\arctan\left(\alpha \right)\right)\right) \end{array}$$ (30)

and

$$\begin{array}{c} x_{i}=Q\left(u_{i}\right) \end{array}$$ (31)

respectively, where *u*_*i*_ with *i* = 1, 2, …, *n* are *n* uniform [0, 1] random numbers, arctan denote the inverse function of trigonometric tangent and *G*^−1^(⋅) is the inverse of *G*(⋅). Note that the model consist of additional parameter *α* > 0 and the support is *a* ≤ *x* ≤ *b* where *a* and *b* take the support of parent distribution.

Consider a Weibull distribution with pdf

$$\begin{array}{cc} g\left(x\right)=\frac{\beta e^{-{\left(\frac{x}{\lambda }\right)}^{\beta }}{\left(\frac{x}{\lambda }\right)}^{\beta -1}}{\lambda }, & \;x>0 \end{array}$$ (32)

In what follows, we illustrate simulation of the arc tan distribution with parent distribution Eq (32). For simplicity, we let *β* = 2 and *λ* = 0.5. The empirical biases and mean square errors of *α* for the Weibull arc tan distribution can be obtained as follows:

1. Ten thousand sample sizes are generated by inversion of Eq (29). Each sample size is *n*. The variates of the Weibull arc tan distribution can be written as
  $$\begin{array}{c} X=\log\left(\frac{1}{2}\sqrt{-\log\left(\frac{\tan\left((1-u)\arctan(\alpha )\right)}{\alpha }\right)}\right) \end{array}$$ (33)
  where *U* ∼ *U*(0,1) is a variates of the uniform distribution.
2. Estimate the parameter, $\hat{\alpha_{i}}$ for *i* = 1, 2, …, 10000.
3. Bias and mean squared error are obtained using
  $$\begin{array}{c} bias_{\alpha }\left(n\right)=\frac{1}{10000}\sum_{i=1}^{10000}\left({\hat{\alpha }}_{i}-\alpha \right) \end{array}$$ (34)
  and
  $$\begin{array}{c} MSE_{\alpha }\left(n\right)=\frac{1}{10000}\sum_{i=1}^{10000}{\left({\hat{\alpha }}_{i}-\alpha \right)}^{2} \end{array}$$ (35)

The above process is repeated for *n* = 10, 20, …, 1000 with *α* = 1.5. The following commands implement the process:

R> nsim   <- 10000

R> nsiz   <- 100

R> est1   <- matrix(0,nsiz,nsim)

R> mm1  <- matrix(0,nsiz,nsim)

R> bias   <- rep(0,nsiz)

R> mse   <- rep(0,nsiz)

R> ss    <- rep(0,nsiz)

R> alpha  <- 1.5

R> for(j in 1:nsiz){

+   for(i in 1:nsim){

+   x    <- rarctan(j*10, alpha = alpha, spec = “weibull”,

+           arg = c(shape = 2, scale = 0.5))

+   nlogl  <- function(p, alpha, spec, arg){

+   tt   <- 1.0e20

+   if(all(p>0)){

+    tt   <- -sum(log(darctan(x, alpha, spec, arg)))

+  }

+  return(tt)

+  }

+   est   <- nlm(function(p) nlogl(p, alpha = p, spec = “weibull”,

+          arg = list(shape = 2, scale = 0.5)), p = 1, hessian = T)

+   est1[j,i]  <- est $estimate [1]

+   mm1[j,1]  <- solve(est $hessian)[1, 1]

+   bias[j]   <- mean(est1[j,]—alpha)

+   mse[j]   <- mean((est1[j,]—alpha)^ 2)

+   ss[j]   <- mean(mm1[j,]^ (1/2))

+ }

#### Analysis of Application on Arc Tan Model

**Fig 6:**
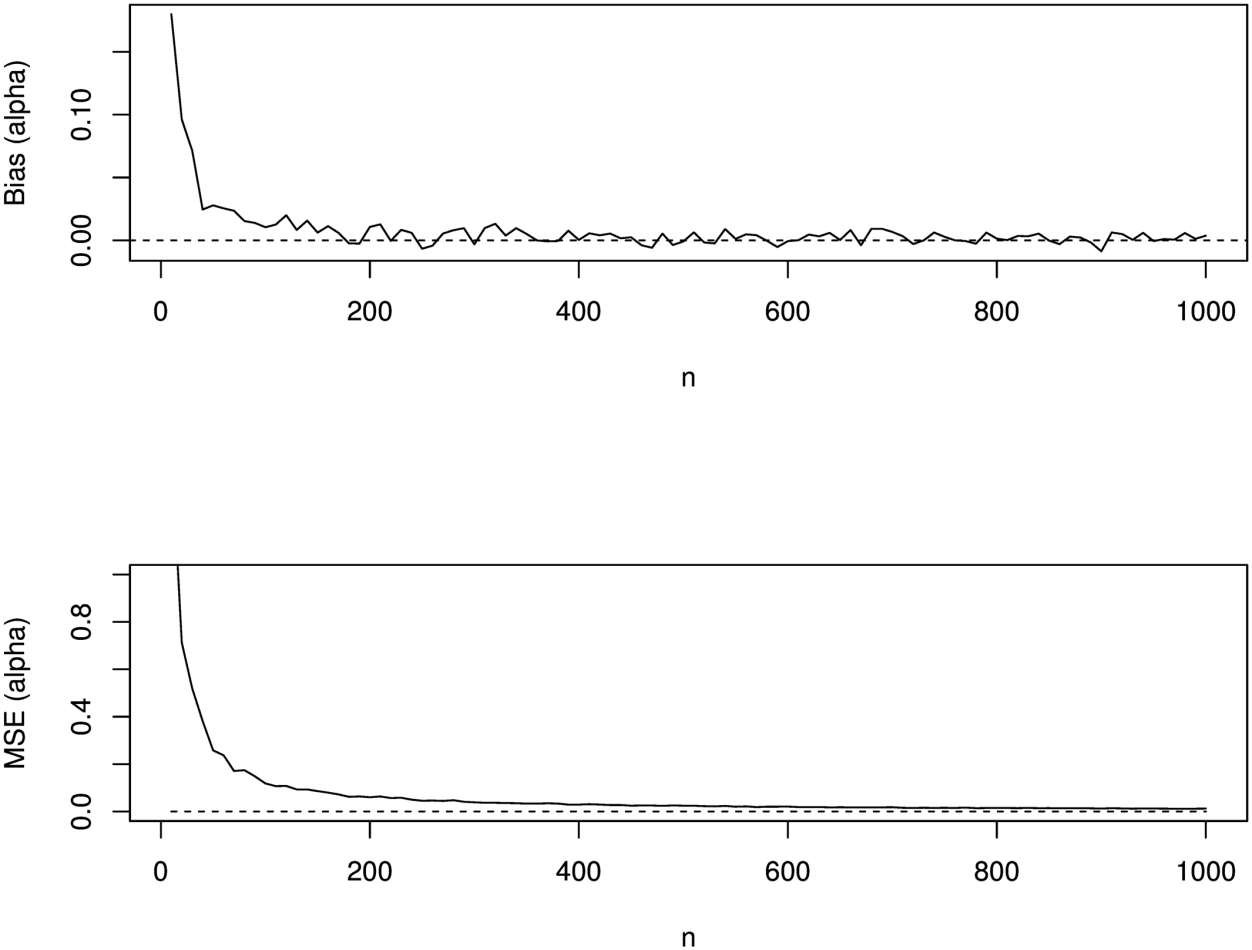
Empirical and theoretical biases and mean square errors of $\hat{\alpha }$ versus *n* = 10, 20, …, 1000. Biases and mean squared errors for Weibull arc tan models are presented for simulated data. The biases are generally positive and decreases to zero as *n* approaches infinity. Similarly, mean squared errors decreases to zero as *n* approaches infinity.

Fig 6 shows the variation of biases and mean squared error with respect to *n*. The dashed line corresponds to zero biases or theoretical mean squared errors, whichever applicable. Several observations can be made:

1. the biases are generally positive
2. as expected, the magnitude of bias always decreases to zero as *n* → ∞
3. the biases are small for $\hat{\alpha }$
4. as expected, the mean squared errors always decrease to zero as *n* → ∞
5. the theoretical mean squared errors are always larger than empirical ones

Results are presented only for *α* = 1.5 due to reason of space. However, similar results can be obtained for other choices of *α*.

An advanced example featuring empirical data related to air quality is given in the Appendix (S1 Appendix). The subroutines provided there emphasize on calling functions from the **gendist** package and thus avoid creating separate object running similar computation. The underlying concept pertaining to this idea make use of do.call which is discussed next.

## Results and Discussion

In this paper, we specify the generated probability distribution models by its parent distribution, that is, the underlying function which generates new models. Codes for writing functions in **gendist** package use a general construct of parent distribution of the form

do.call(paste(d, spec, sep = ""), c(list(z), arg)),

do.call(paste(d, spec1, sep = ""), c(list(z), arg1))

or

do.call(paste(d, spec2, sep = ""), c(list(z), arg2)).

As such, spec = “Weibull”, for instance, corresponds to Weibull parent distribution and arg = list(shape, scale) list its parameters. Similarly, for two component distributions spec1 and spec2 specify the parent distributions with corresponding parameters arg1 and arg2.

The parent distribution may assume any value on real line or positive real numbers. The suitability of the parent distributions for the models must be checked by the user. No warnings are given for choosing inappropriate distributions. Table 1 describes a proper selection for the parent distribution corresponding to each model with respect to its support.

**Table 1:** Support for models in **gendist** package.

Models	Support of parent distribution	Support of generated models
Mixture	Real numbers and positive real numbers	Real numbers and positive real numbers
Composite	Real numbers and positive real numbers	Real numbers and positive real numbers
Folded	Real numbers	Positive real numbers
Skewed	Real numbers	Real numbers
Arc tan	Real numbers and positive real numbers	Real numbers and positive real numbers

Functions to compute pdf, cdf, qf and rg for all five models produced in **gendist** are summarised in Table 2. As mentioned earlier, the input argument spec, spec1 and spec2 specify the parent distribution and their corresponding parameters arg, arg1 and arg2. The distribution can be one that is implemented in R **base** package, contributed R packages or one written by a user. In any case, the parent functions must be defined with prefix d, p, q and r attached to spec, spec1 and spec2.

**Table 2:** Calling sequences for mixture, composite, folded, skewed symmetric and arc tan models.

Models	Functions	Calling sequence
Mixture	*f*(*x*) in Eq (2)	dmixt(x, phi, spec1, arg1, spec2, arg2, log = FALSE)
	*F*(*x*) in Eq (3)	pmixt(q, phi, spec1, arg1, spec2, arg2, lower.tail = TRUE, log.p = FALSE)
	*Q*(*x*) in Eq (4)	qmixt(p, phi, spec1, arg1, spec2, arg2, interval = c(0,100), lower.tail = TRUE, log.p = FALSE)
	*x*_*i*_ in Eq (5)	rmixt(n, phi, spec1, arg1, spec2, arg2, interval = c(0,100))
Composite	*f*(*x*) in Eq (6)	dcomposite(x, spec1, arg1, spec2, arg2, initial = 1, log = FALSE)
	*F*(*x*) in Eq (9)	pcomposite(q, spec1, arg1, spec2, arg2, initial = 1, lower.tail = TRUE, log.p = FALSE)
	*Q*(*x*) in Eq (10)	qcomposite(p, spec1, arg1, spec2, arg2, initial = 1, lower.tail = TRUE, log.p = FALSE)
	*x*_*i*_ in Eq (11)	rcomposite(n, spec1, arg1, spec2, arg2, initial = 1)
Folded	*f*(*x*) in Eq (14)	dfolded(x, spec, arg, log = FALSE)
	*F*(*x*) in Eq (13)	pfolded(q, spec, arg, lower.tail = TRUE, log.p = FALSE)
	*Q*(*x*) in in Eq (16)	qfolded(p, spec, arg, interval = c(0,100), lower.tail = TRUE, log.p = FALSE)
	*x*_*i*_ in Eq (17)	rfolded(n, spec, arg, interval = c(0,100))
Skew symmetric	*f*(*x*) in Eq (21)	dskew(x, spec1, arg1, spec2, arg2, log = FALSE)
	*F*(*x*) in Eq (22)	pskew(q, spec1, arg1, spec2, arg2, lower.tail = TRUE, log.p = FALSE)
	*Q*(*x*) in Eq (22)	qskew(p, spec1, arg1, spec2, arg2, interval = c(1,10), lower.tail = TRUE, log.p = FALSE)
	*x*_*i*_ in Eq (24)	rskew(n, spec1, arg1, spec2, arg2, interval = c(1,10))
Arc tan	*f*(*x*) in Eq (28)	darctan(x, alpha, spec, arg, log = FALSE)
	*F*(*x*) in Eq (29)	parctan(q, alpha, spec, arg, lower.tail = TRUE, log.p = FALSE)
	*Q*(*x*) in Eq (30)	qarctan(p, alpha, spec, arg, lower.tail = TRUE, log.p = FALSE)
	*x*_*i*_ in Eq (31)	rarctan(n, alpha, spec, arg)

It is important to note that some models may not have explicit general form for its probability functions. Whenever this is the case, numerical methods are used. In **gendist**, we utilise integrate, nlm and uniroot functions. Some of these functions, in particular, uniroot require interval values to search for roots and they are specified by interval. nlm require a starting value specified by initial.

## Conclusions

In this paper, we discuss five models provided in the R package gendist, namely, the mixture model, the composite model, the folded model, the skewed symmetric model and the arc tan model. The functions related to these models are written in R environment and are freely downloadable from http://www.r-project.org, see [33]. Users can easily create any specific model by specifying the parent distributions along with their parameters. Thus, uncountable number of distributions can be produced from the tools in the **gendist** package. Another advantage is that the users have the option to write their own function to serve as the parent distributions.

Next, we also show at least five important applications of the tools given in the **gendist** package. Usefulness of these tools in graphing are shown for Q-Q plot, P-P plot, producing pdf curves as well as to show output for some risk measures. Simulation study is provided for a specific Weibull arc tan model and produced favorable result with respect to biases and mean squared error of the parameter estimated. Both measures decrease to zero when the number of observations approaches infinity.

Nevertheless, we only provide pdf, cdf, qf and rg for five common models that recently receive applications in actuarial loss modeling. Some of the models have also been considered in other areas while others such as arc tan model are potentially useful in areas involving parametric framework. Many other available models can be designed in similar general form as the **gendist** package and thus avoid the problem of offering separate R packages for distributional models of the same class into production of a single package that incorporates all. Therefore, we encourage other scientists to furnish the package with additional models in their respective field or send suggestion for further improvement. The **gendist** package provides a great flexibility to work with many distributions and hopes to assist users in their respective fields.

## Supporting Information

S1 Script Script to replicate examples. The script to replicate examples in this paper is provided in (.R) form.
(R) Click here for additional data file.

S1 Appendix Appendix. (TXT)

S1 Data Loss data. (TXT)

S2 Data Rivers Data. (TXT)

S3 Data Air Quality Data. (TXT)
