# Correction: Gendist: An R Package for Generated Probability Distribution Models

**DOI:** 10.1371/journal.pone.0160903

**Published:** 2016-08-04

**Authors:** The PLOS ONE Staff

In the title of this article, the word “Gendist” should be “gendist”. The correct title is: gendist: An R Package for Generated Probability Distribution Models. The correct citation is: Abu Bakar SA, Nadarajah S, ABSL Kamarul Adzhar ZA, Mohamed I (2016) gendist: An R Package for Generated Probability Distribution Models. PLoS ONE 11(6): e0156537. doi:10.1371/journal.pone.0156537. The publisher apologizes for the error.
